# Study of the potential of composite material based on zeolite and geopolymer as an adsorbent of toxic metals

**DOI:** 10.1007/s11356-026-37751-7

**Published:** 2026-04-29

**Authors:** Ricardo Jadson da Silva Nascimento, Inácio Cruz de Loiola, João Vitor Torres Sousa, Vicente de Oliveira Sousa Neto, Luiz Thiago Vasconcelos da Silva, Ronaldo Ferreira do Nascimento

**Affiliations:** 1 Laboratory of Trace Analysis-LAT, Department of Analytical and Physical Chemistry, Federal University of Ceara, Fortaleza, Brazil; 2 Laboratory of Study and Research in Pollutants Removal By Adsorption-LERPAD, Department of Chemistry, State University of Ceara, Quixadá, Brazil

**Keywords:** Adsorption, Geopolymer, Zeolite A, Fly ash, Heavy metal

## Abstract

The uncontrolled disposal of effluents generated by the electroplating industry represents significant environmental challenges due to the toxicity and persistence of toxic metals present. The present study evaluated a geopolymer-zeolite A composite adsorbent, synthesized from fly ash of steel mill furnaces, for the removal of Cu^2+^, Ni^2+^, and Zn^2+^ from aqueous solutions. The produced material exhibited properties (structural and morphological) of both components, which were confirmed by physicochemical characterizations. Batch adsorption studies were performed in a synthetic solution (simulating a galvanic effluent). Kinetic studies revealed satisfactory adsorption rates for Cu^2^⁺ and Zn^2^⁺, while Ni^2^⁺ showed low performance. The experimental adsorption isotherm data were best described by the Sips model, indicating the heterogeneity of the material’s surface. The maximum adsorption capacities were 162,393 mg·g⁻^1^ (Zn^2^⁺), 99,801 mg·g⁻^1^ (Cu^2^⁺), and 63,266 mg·g⁻^1^ (Ni^2^⁺). The removal efficiencies relative to the initial concentration (0.8 mmol L⁻^1^) were 34.7% (Cu^2+^), 32.67% (Zn^2+^), and 17.12% (Ni^2+^). Textural analysis revealed that the composite exhibits intermediate behavior between the base phases, with an increased surface area (SBET = 7 m^2^·g^−1^) and pore volume, which enhances active site accessibility, while FTIR spectra confirmed the structural integrity of the aluminosilicate framework post-adsorption. The dominant mechanism of ion exchange was verified, due to the substantial increase in Na^+^ concentration (from 800 to 1112 mg·L^−1^). The results suggest that the developed composite is a promising route for valorizing legacy coal fly ash, demonstrating strong adsorptive properties for specific heavy metals in simulated aqueous systems. **Clinical trial number**: Not applicable.

**Supplementary information:**

The online version contains supplementary material available at https://doi.org/10.1007/s11356-026-37751-7.

## Introduction

The demand for natural resources and intense industrial activity have strongly contributed to increased environmental degradation, particularly water pollution. Water, essential for life and numerous human activities, has been compromised by the introduction of anthropogenic contaminants, affecting aquatic ecosystems, public health, and global water security (United Nations Children’s Fund (UNICEF) and World Health Organization (WHO), 2023). Although technological advances have improved living conditions, they have also driven an unsustainable socio-economic model, increasing the pressure on natural resources (Santos et al. 2024; F. Wang et al. 2023).

Among the most concerning pollutants are toxic metals such as copper (Cu), nickel (Ni), zinc (Zn), lead (Pb), and cadmium (Cd), which exhibit high toxicity, environmental persistence, and bioaccumulation potential, even at low concentrations (Angon et al. 2024; Campillo-Cora et al. 2025). Their presence in aquatic environments alters biodiversity, reduces reproduction rates of organisms, and compromises food security (Angon et al. 2024; Jomova et al. 2025). Among the industrial sources of these pollutants, electroplating stands out as an electrochemical process widely used in metal surface coating but constitutes a significant source of effluents contaminated with toxic metals (Oladimeji et al. 2024; Peng et al. 2020; Rajoria et al. 2022).

In light of these risks, national and international regulations have established increasingly stringent limits for the disposal of wastewater containing toxic metals (Jomova et al. 2025). This reinforces the need to develop more efficient, economically viable, and environmentally sustainable industrial wastewater treatment technologies (Oladimeji et al. 2024; Rajoria et al. 2022). In this context, the adsorption technique emerges as a promising alternative, combining operational simplicity, low cost, and high efficiency in contaminant removal (Meftah et al. 2025; Rajoria et al. 2022; Xu et al. 2021). Recent research reviews confirm that inorganic adsorbents, such as zeolites and geopolymers, exhibit competitive performance in removing toxic metals; however, challenges, including selectivity, regenerability, and cost, still need to be addressed (Adewuyi 2021; Hamzah et al. 2025; Ren et al. 2022).

The efficiency of an adsorptive process depends on the characteristics of the adsorbent. Zeolites (crystalline aluminosilicates) with a three-dimensional and tetrahedral structure (interconnected by oxygen), possess a network of channels and cavities that provide cation exchange interaction and selective retention of solutes (da Silva et al. 2023; Indira & Abhitha 2023; Ren et al. 2022). Geopolymers, on the other hand, are inorganic polymers obtained through the alkaline activation of aluminosilicate materials, forming amorphous matrices with high mechanical strength, durability, and chemical stability (Adewuyi 2021; Ren et al. 2022). Recent studies have shown that fly ash-derived geopolymers achieve adsorption capacities exceeding 90 mg/g for Cu^2^⁺ and Zn^2^⁺, highlighting their applicability in real systems (Darmayanti et al. 2024). Hybrid materials made of zeolite and geopolymer have also attracted interest, as they offer the potential to explore structural and chemical synergies. For example, composites prepared with a high zeolite content have achieved removal rates of up to 446 mg/g for Pb^2+^ in aqueous solution (Yang et al. 2022).

Moreover, the production of zeolites and geopolymers from industrial waste, such as fly ash and slags, has been considered a sustainable and low-cost strategy aligned with the circular economy (Adewuyi 2021; Ren et al. 2022). Recent advancements have shown that the in situ hydrothermal synthesis of zeolite-geopolymer composites yields porous materials with high surface areas and chemical stability, thereby expanding their applicability in adsorption processes (Adewuyi 2021; Li et al. 2025).

Several studies have demonstrated the effectiveness of synthetic materials derived from industrial waste. However, most existing studies are limited to single-metal adsorption systems, failing to consider the competitive dynamics of multi-element matrices. Furthermore, these studies often demonstrate an inability to simulate or predict the complex multi-element profiles and high metal concentrations characteristic of real industrial effluents. Moreover, there is a lack of research on the application of geopolymer-zeolite composites in the treatment of wastewater contaminated with various sets of toxic metals typically found in galvanic effluents (Luhar & Luhar 2022; Thakur et al. 2022).

The long-term viability of these materials should be evaluated within a circular economy framework. High-performance adsorbents, such as chitosan derivatives and synthetic zeolites, are limited by costly and complex synthesis (da Silva Alves et al. 2021; Eren et al. 2024). In addition, low-cost polymeric adsorbents may pose environmental risks through microplastic release (Bodzek et al. 2024; Joo et al. 2021). Although raw coal fly ash (CFA) can lead to toxic metals leaching, geopolymerization immobilizes hazardous elements within a stable three-dimensional structure, enhancing environmental safety (Luhar & Luhar 2022; Toniolo & Boccaccini 2017). Considering the global energy transition and the significant CFA generation in regions such as Ceará, Brazil (da Silva et al. 2023; Santos et al. 2024), CFA-based technologies must anticipate reduced coal dependency. Therefore, the geopolymer–zeolite synthesis routes proposed here are adaptable to alternative precursors, including blast furnace slag and calcined clays (Provis 2014; Maged et al. 2023; Nodehi & Taghvaee 2022; Sarkar et al. 2017), ensuring the continued relevance of ultra-low-cost adsorbents in a post-coal circular economy.

The integration of zeolite and geopolymer phases offers a promising strategy for valorizing coal fly ash in low-cost adsorbents. This study evaluates the preliminary performance of geopolymer-zeolite A composites in removing toxic metals from simulated industrial effluents under high load conditions, including studies of material characterization, kinetics, and adsorption isotherms.

## Material and methods

### Chemical reagents

The reagents and analytical standards used in the adsorption study are described below: Ni(NO_3_)_2_·6H_2_O (VETEC, Brazil, purity ≥ 98%), NiSO_4_·6H_2_O (VETEC, Brazil, purity ≥ 98%), ZnSO_4_·7H2O (VETEC, Brazil, purity ≥ 98%), CuSO_4_ (VETEC, Brazil, purity ≥ 98%), CuCl_2_·2H_2_O (SIGMA-ALDRICH, Brazil, purity ≥ 98%), KNO_3_ (NEON, Brazil, purity ≥ 97%), CH_3_COONa (EXODUS SCIENTIFIC, USA, purity ≥ 99%), glacial acetic acid (EXODUS SCIENTIFIC, USA, purity ≥ 99%) and deionized water (Milli-Q).

### Composite synthesis

**Fig. 1:**
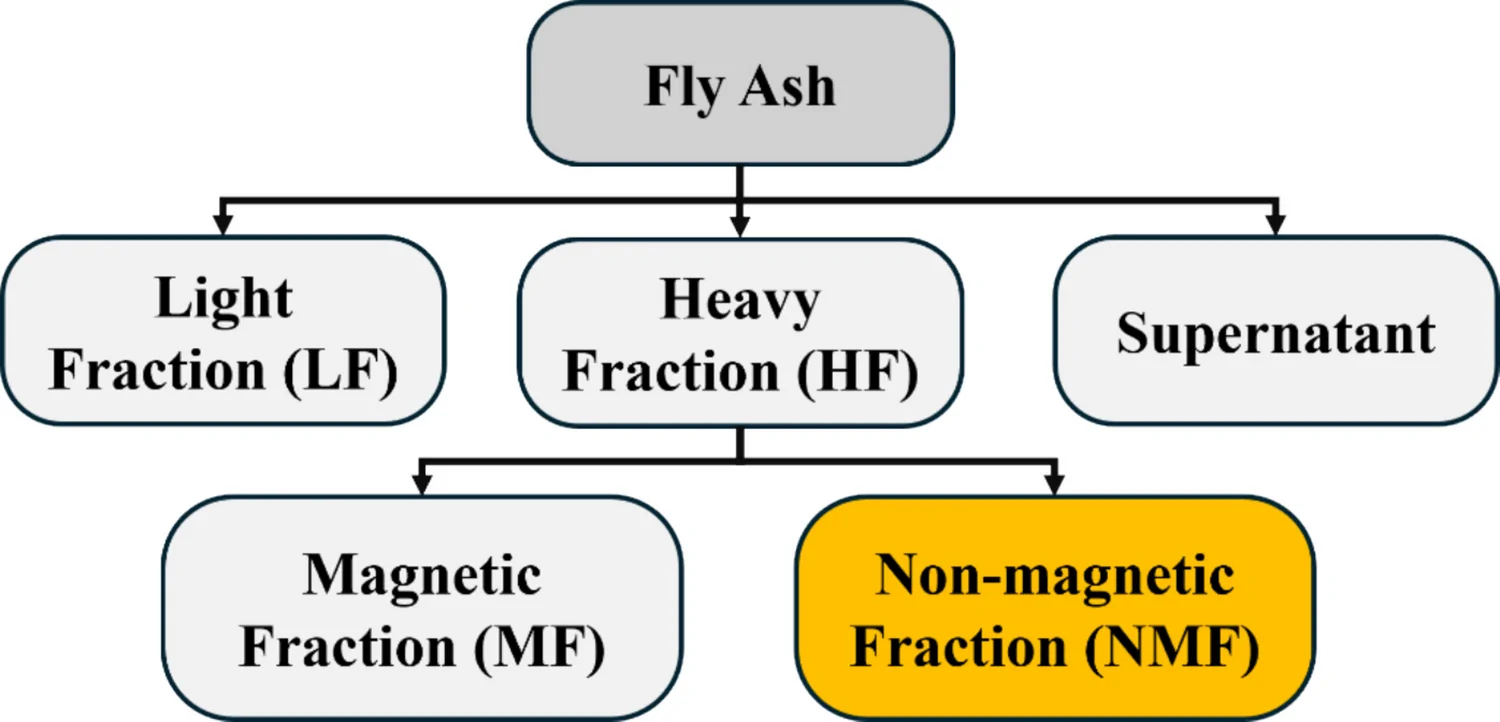
Flowchart of the process for obtaining the fractions of fly ash

The composite material was prepared by mixing zeolite A and expanded geopolymer. The geopolymer was prepared from fly ash, derived from coal combustion (non-magnetic fraction, NMF), containing on average 77% of the total ash mass. The fraction was obtained according to the scheme shown in Fig. 1. The ash sample is stirred in an aqueous medium for 24 h, generating three portions: the dissolved fraction (supernatant), the suspended fraction (light fraction—LF), and the sedimented fraction (heavy fraction—HF). The HF fraction is separated magnetically, and the result is the non-magnetic fraction (NMF) used as a precursor for the synthesis of the geopolymer.

The zeolite was synthesized by adding 5 g of metakaolin to 60 mL of 2.8 mol·L^−1^ NaOH solution until complete homogenization. After, the mixture is left to stand for 18 h at room temperature and then placed in a Teflon® capsule in an oven at 100 °C for 4 h. At the end, the material was washed (with distilled water) until pH < 9.0.

The preparation of the composite involved mixing 18 g of NMF, 2 g of metakaolin and 0.6 g of zeolite A. The solid material was homogenized dry and then were added 10 mL of 1.0 mol L^−1^ NaOH and 10 mL of H₂O₂ were added. Then, it was homogenized again, transferred to Teflon® capsules and subjected to heat treatment in an oven at 90 °C for 28 days. At the end, the solid obtained was ground, washed with distilled water (to remove soluble residues) and dried in an oven.

### Material characterization

#### X-ray diffraction (XRD)

Previously, the samples were ground into a fine powder and mounted in a standard stainless steel sample holder. XRD identified the crystalline phases in the samples and the degree of crystallinity of the solids. XRD results were obtained at room temperature using a Panalytical diffractometer. Scans were performed in the range of 10 to 80° (2θ), with a step of 0.013° and a scan speed of 0.5 s/step.

#### Scanning electron microscopy (SEM)

The samples were previously mounted on aluminum supports using double-sided carbon tape and coated with a thin layer of gold in an argon atmosphere and under low pressure, using a Q15DT ES sputtering metallizer (Quorum). SEM evaluated the surface morphology and microstructure of the materials. SEM results were obtained using a Quanta FEG 450 scanning electron microscope (FEI), operating with an electron beam accelerated between 5.0 and 20.0 kV, equipped with secondary electron detectors (SED) and energy-dispersive X-ray spectroscopy (EDS).

#### Fourier transform infrared spectroscopy (FTIR)

Previously, the samples were dispersed in potassium bromide (KBr) and pressed into pellets. FTIR was used to identify the functional groups present in the samples and reveal evidence of relevant surface interactions in the adsorption process studied. The analyses were performed in the spectral range of 4000 to 400 cm⁻^1^ using a Shimadzu IRTracer-100 spectrometer.

#### ***pH zero-point of charge (pH***_***pzc***_***)***

The pHpzc of the adsorbent was determined using the adapted methodology known as the 11-point method (Postai et al. 2016; Santos et al. 2024), as described: Eleven 0.01 mol·L⁻^1^ NaCl solutions were prepared, with the pH adjusted to values between 1 and 11, using 0.1 mol·L⁻^1^ HCl and NaOH solutions. Then, 50 mL aliquots of each solution were transferred to Erlenmeyer flasks containing 50 mg of the adsorbent (in triplicate). The solutions were stirred at 220 rpm for 24 h at room temperature. After this time, the samples were filtered and the final pH of the solutions was measured. The pHpzc was estimated graphically (initial pH versus final pH), considering the average value of the points in the plateau region, where the final pH remained practically constant.

#### X-ray fluorescence (XRF)

X-ray fluorescence (XRF) is an analytical technique based on the measurement of the intensity of characteristic X-rays emitted by the constituent elements of a sample, allowing the determination of its chemical composition. XRF analyses were performed using a sequential wavelength-dispersive X-ray spectrometer (WDX) from Rigaku, model ZSX Mini II, operated at 40 kV and 1.2 mA and equipped with a palladium (Pd) X-ray tube. The instrument enables semiquantitative analysis of elements with atomic numbers equal to or higher than that of fluorine up to uranium.

### Batch adsorption study

#### Characterization of the galvanic effluent and preparation of the synthetic solution

A sample of galvanic effluent was provided by a company located in the city of Juazeiro do Norte, Ceará, Brazil, which was used as a reference for the preparation of the aqueous solution studied. A metal electroplating company provided the industrial effluent sample used as a reference in this study.

In order to identify the main constituents and determine key parameters for the design of adsorption experiments, the effluent pH was measured, the concentrations of the major anions were quantified using an ion chromatograph (Dionex ICS 5000, Thermo Scientific), while the concentrations of the main metallic cations, such as Cu^2+^, Ni^2+^, and Zn^2+^, were determined by flame atomic absorption spectrometry (FAAS) using an AA 240 FS spectrometer (Varian). Based on the characterization of the collected sample, a synthetic aqueous solution of the effluent was prepared with the addition of previously identified and quantified cations and anions. The concentrations of the metallic cations were set at 5 mmol L⁻^1^ to evaluate their competitive adsorption behavior. The concentrations of the anions (chloride, sulfate, and nitrate) were maintained at levels close to those found in the real effluent. The pH of the synthetic solution was adjusted to 5.0 with a buffer solution (acetic acid/sodium acetate). The analyses were performed in triplicate to ensure the reliability and reproducibility of the results.

#### Dosage test

The dosage experiments were conducted in triplicate, using 50 mL of synthetic effluent, with adsorbent mass varying from 10 to 100 mg. The solutions were stirred at 220 rpm for 24 h at room temperature. After that, the supernatant was collected and filtered, and the residual concentration of each metal ion was determined by FAAS.

#### Adsorption kinetics

The adsorption kinetics experiments were performed involving three equimolar concentrations for a multi-element solution of the metal ions: 5.0, 2.5, and 0.8 mmol·L⁻^1^, keeping the anion concentration fixed in all solutions. The solution was stirred at 220 rpm for 3 h at room temperature. All experiments were performed in triplicate. Aliquots were collected (filtered) at predetermined time intervals to determine the concentration of metal ions by FAAS over time. The experimental data were investigated by kinetic models (in their non-linear forms): pseudo-first order (PFO), Eq. (1) (Lagergren 1898); pseudo-second order (PSO), Eq. (2) (Ho & McKay 1999); and the Elovich model, Eq. (3) (McLintock 1967).

where: qₜ is the amount of metal ion adsorbed at time t (mg·g⁻^1^), qₑ is the amount adsorbed at equilibrium (mg·g⁻^1^), k₁ (min⁻^1^) is the rate constant of the PFO model, and k₂ (g·mg⁻^1^·min⁻^1^) is the rate constant of the PSO model. In the Elovich model, α (mg·g⁻^1^·min⁻^1^) is the initial adsorption rate, and β (g·mg⁻^1^) is the constant related to the extent of surface coverage and the activation energy of chemisorption.

1 $${q}_{t}={q}_{e}(1-{e}^{{-k}_{1}t})$$

2 $${q}_{t}=\frac{{q}_{e}^{2}{k}_{2}t}{1+{q}_{e}{k}_{2}t}$$

3 $${q}_{t}=\frac{1}{\beta }ln \left(1+\alpha \beta t\right)$$

#### Adsorption isotherm

The study of adsorption isotherms was carried out using equimolar multielement solutions (Cu^2^⁺, Ni^2^⁺ and Zn^2^⁺). The initial concentrations of the metal ions ranged from 0.8 to 5.0 mmol·L⁻^1^. The equilibrium time adopted was established based on the kinetic study, and the residual concentration of each metal ion was determined according to the experimental condition described previously in the kinetic study. The experimental data were fitted to the Langmuir isotherm model (Eq. (4)) (Langmuir 1918), the Freundlich model (Eq. (5)) (H. M. F. Freundlich 1906), and the Sips model (Eq. (6)) (Sips 1948), allowing for the estimation of key parameters such as the maximum adsorption capacity, the affinity between adsorbate and adsorbent, and the degree of heterogeneity of the adsorbent surface.

The Langmuir isotherm model (Eq. (4)) assumes that adsorption occurs at specific, energetically equivalent, and uniformly distributed sites, forming a monolayer with a thickness of a single molecule (González-López et al. 2022; Y. Wang et al. 2024).

The Freundlich isotherm model (Eq. (5)), in contrast, does not assume adsorbent saturation, implying that surface coverage can increase indefinitely, and suggesting the formation of multilayer adsorption (Y. Wang et al. 2024).

The Sips isotherm (Eq. (6)) is a hybrid model that combines features of both Langmuir and Freundlich models, aiming to describe heterogeneous adsorption systems with a finite adsorption capacity (Santos et al. 2024; Sips 1948; Y. Wang et al. 2024).

4 $${q}_{e}=\frac{{q}_{max}{K}_{L}{C}_{e}}{1+{K}_{L}{C}_{e}}$$

5 $${q}_{e}={K}_{F}{C}_{e}^{1/n}$$

6 $${q}_{e}=\frac{{q}_{max}{({K}_{S}{C}_{e})}^{n}}{1+{({K}_{S}{C}_{e})}^{n}}$$

where for equation of Langmuir, qₑ is the amount of solute adsorbed at equilibrium (mg·g⁻^1^), Cₑ is the equilibrium concentration of the metal ion in the solution (mg·L⁻^1^), qₘₐₓ is the maximum adsorption capacity (mg·g⁻^1^) and K_L_ is the Langmuir constant (L·mg⁻^1^). For equation of Freundlich, K_F_ is the Freundlich constant related to adsorption capacity [(mg·g⁻^1^) (L·mg⁻^1^)^1^⁄ⁿ], and *1/n* is a dimensionless parameter indicative of adsorption intensity and surface heterogeneity. For the Sips model, *K*_*S*_ is the Sips equilibrium constant (L·mg⁻^1^), and *n* is the heterogeneity factor that reflects the system’s deviation from ideal homogeneous adsorption.

#### Adsorption mechanism

The porosity and area of the materials were determined using argon (Ar) adsorption/desorption isotherms at − 196 °C, in a relative pressure (P/Po) range of 0.01 to 0.995 atm. Measurements were performed using a volumetric adsorption analyzer (Micromeritics ASAP 2020 Plus). Previously, the samples were degassed (at 300 °C for 8 h) to remove volatile impurities. The specific surface area (SBET) was calculated using the BET (Brunauer–Emmett–Teller) method, and the total pore volume was calculated at the point of maximum relative pressure. Regarding the ion exchange mechanism speculated in the adsorption process, a specific cation release assay was conducted to prove it. The adsorbent mixture was prepared with the synthetic solution of metal ions (5 mmol L⁻^1^), in triplicate, under constant stirring at 220 rpm and a controlled temperature of 25 °C, for a contact equilibrium time of 3 h, as previously determined in the kinetics study. After this, the supernatant (filtrate) was separated and the Na⁺ concentration in the filtrate was determined by flame photometry. The results were compared (before and after adsorption) to evaluate the displacement of charge compensation cations from the aluminosilicate matrix by the target heavy metal ions (Cu^2^⁺, Ni^2^⁺, and Zn^2^⁺). Finally, the structural stability and potential surface interactions were evaluated by Fourier Transform Infrared Spectroscopy (FTIR), before and after the adsorption process. The spectra were recorded in the 4000–400 cm^−1^ range, allowing the identification of changes in the functional groups and in the integrity of the geopolymer-zeolite structure.

## Results and discussion

### Material characterization

#### Scanning electron microscopy (SEM)

**Fig. 2:**
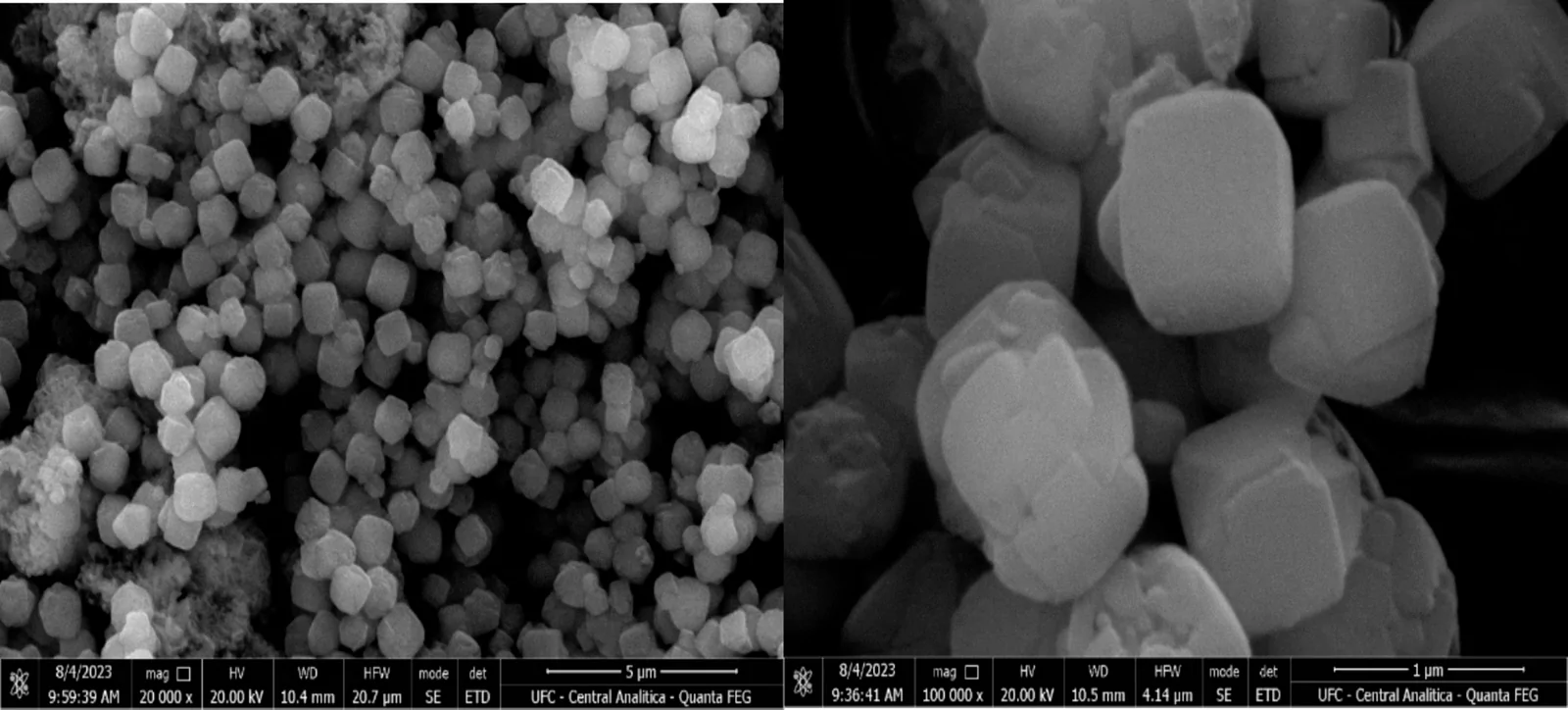
Micrograph of Zeolite A

The SEM micrographs obtained for the synthesized zeolite A sample (Fig. 2) reveal typical structural characteristics of consistent morphology expected for this material. The particles exhibit a relatively homogeneous size distribution and appear to be organized in aggregates, possibly resulting from the drying and heat treatment processes subsequent to synthesis. Therefore, substantial alterations in the standard crystalline structure of zeolite A are observed. In contrast to the cubic morphology typically observed in conventionally synthesized samples, the crystals presented more rounded contours, without sharp edges. The aforementioned morphological modifications can be attributed to the particular conditions employed during the synthesis process.

**Fig. 3:**
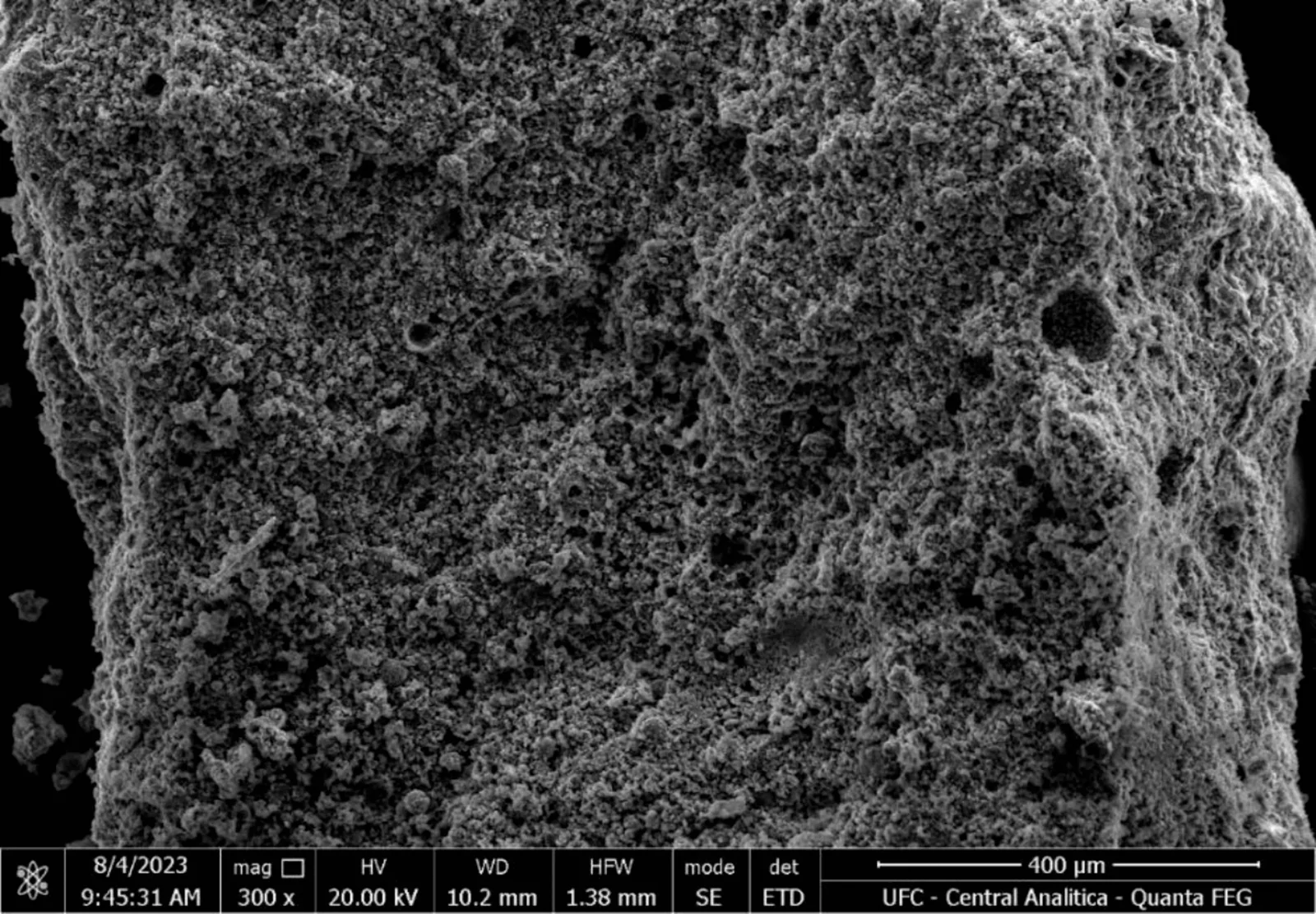
Micrograph of the pure geopolymer

Figure 3 shows the micrograph obtained for the pure geopolymer. The presence of particles with irregular morphology and lack of defined order is evident, typical characteristics of materials with an amorphous structure. On the other hand, regions with a more organized appearance can also be identified, suggesting the presence of semi-crystalline phases. This observation is consistent with the nature of the raw material used, composed mainly of precipitated silica derived from coal fly ash. In addition, the formation of agglomerates composed of small interconnected particles, forming a continuous solid network, can also be observed. This behavior is aligned with the standard polymerization mechanism of geopolymeric materials, as established by studies in the field.

**Fig. 4:**
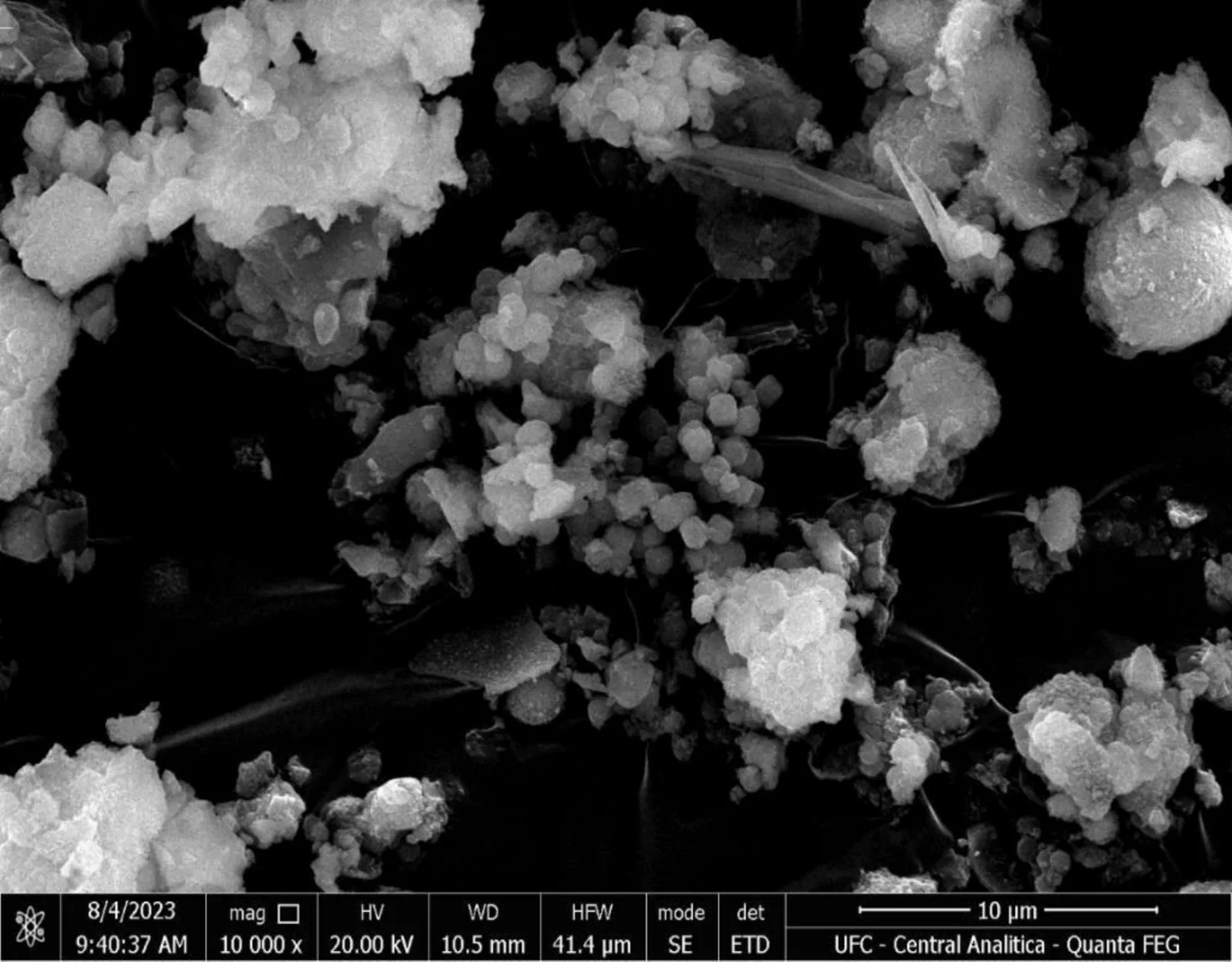
Micrograph of the composite

The micrograph obtained for the composite (Fig. 4) clearly reveals the presence of two distinct phases, evidencing the preservation of the morphological characteristics of both zeolite A and the geopolymer matrix. The amorphous phase, associated with the geopolymer, manifests as a dense matrix with an irregular surface texture, while the crystalline phase, corresponding to zeolite A, is observed as dispersed cubic particles immersed in the matrix. The characteristic morphology of zeolite A was not modified after its incorporation into the composite, indicating that the processes did not compromise its structural integrity. In addition, crystal growth is observed on the surface of the polymer agglomerates, suggesting that the nucleation and development of zeolitic units may have occurred during the composite formation process.

#### X-ray diffraction (XRD)

**Fig. 5:**
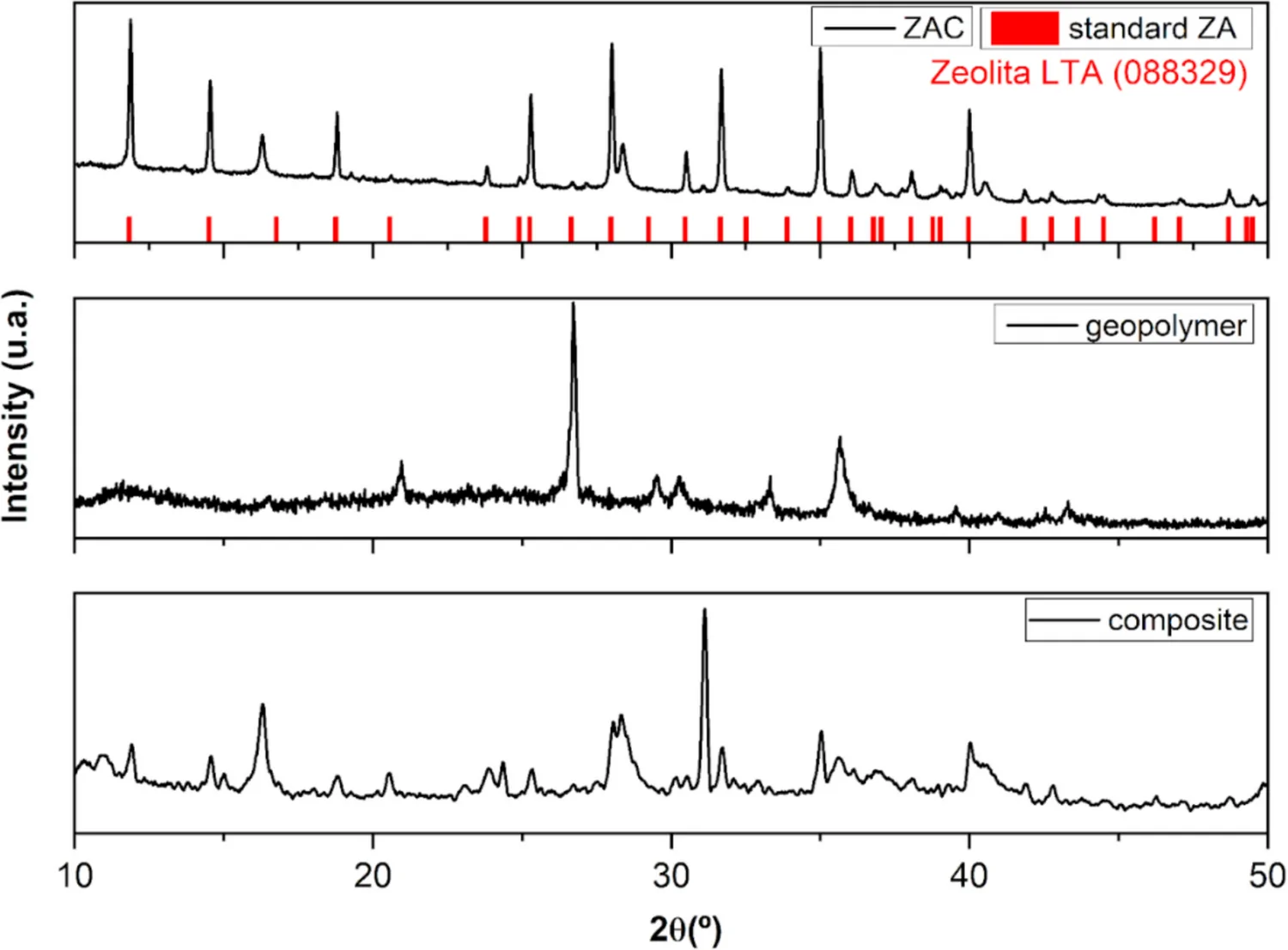
X-ray diffraction patterns of Zeolite A, geopolymer, and zeolite–geopolymer composite obtained using Co Kα radiation (λ = 0.1789 nm) over the 2θ range from 10 to 50°

Figure 5 presents the X-ray diffraction (XRD) patterns of Zeolite A, the geopolymer, and the zeolite–geopolymer composite, allowing a direct comparison of their structural characteristics. The synthesized Zeolite A exhibits intense, sharp, and well-defined diffraction peaks, which are in excellent agreement with the reference pattern of the LTA framework reported by the International Zeolite Association (International Zeolite Association, 2001), confirming the formation of a highly crystalline and well-ordered phase. In contrast, the geopolymer displays a diffraction pattern dominated by a broad amorphous halo between approximately 15° and 40° (2θ), with a maximum around 28°, characteristic of disordered aluminosilicate networks typical of geopolymer materials (Dembovska et al. 2018; Provis et al. 2005). The presence of minor diffraction peaks indicates a semicrystalline nature, likely associated with residual crystalline phases from the precursors. The composite diffractogram combines features of both materials. The amorphous halo of the geopolymer is preserved but with reduced intensity, suggesting a decrease in structural disorder, while distinct peaks attributed to Zeolite A (e.g., at ~ 16°, ~ 32°, and ~ 40°) remain clearly visible. This indicates that the zeolite crystalline structure is retained after incorporation into the geopolymer matrix. Overall, the XRD results demonstrate that the composite exhibits a hybrid structure, consisting of a continuous amorphous geopolymer phase embedding crystalline zeolite domains, in agreement with the SEM morphological observations.

#### Fourier transform infrared spectroscopy (FTIR)

**Fig. 6:**
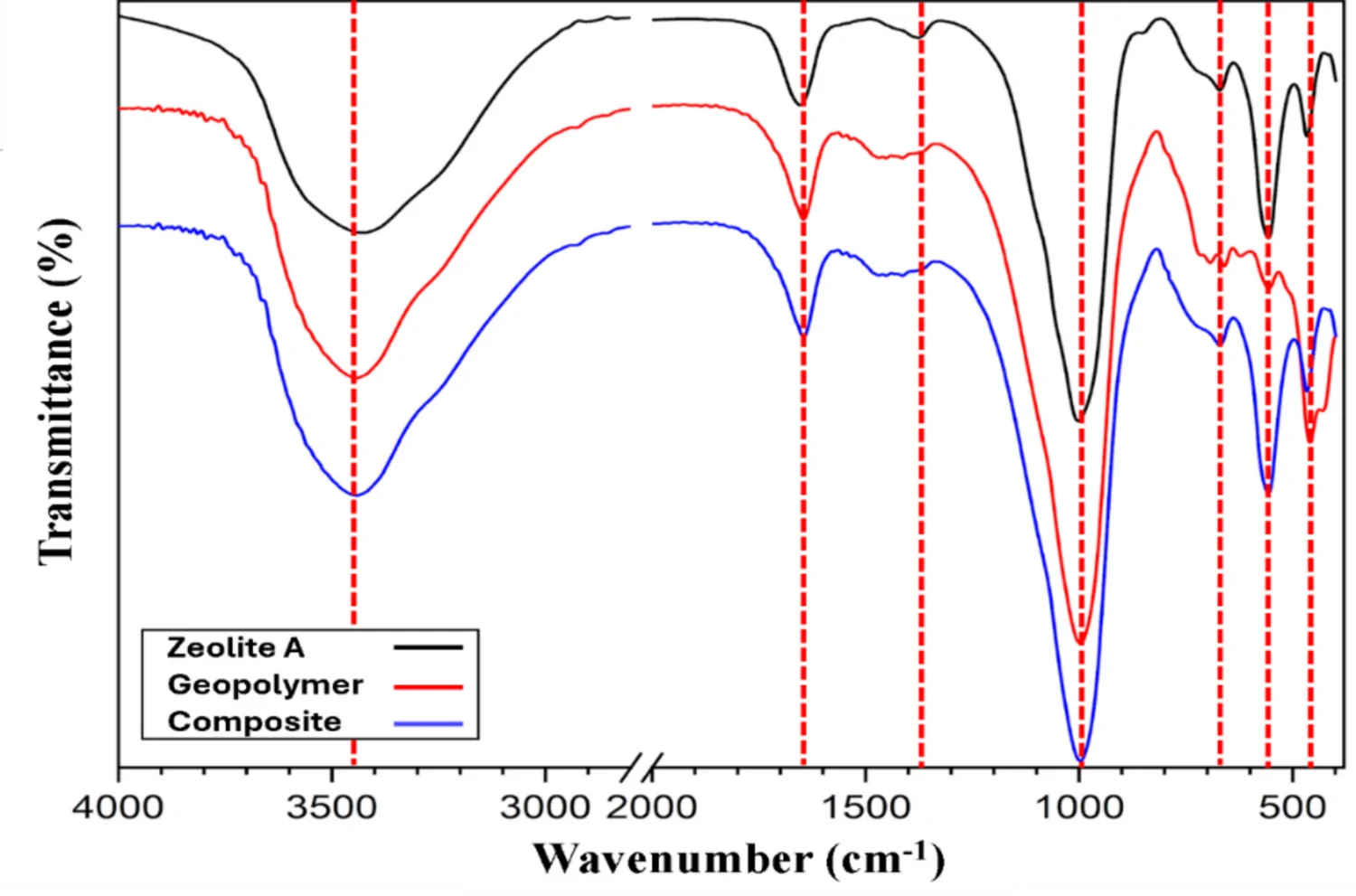
Infrared spectra: (**a**) Zeolite A, (**b**) Geopolymer, (**c**) Composite

Figure 6 shows the comparative infrared spectra of the three materials. The FTIR spectrum of zeolite A exhibits characteristic bands in the 450–750 cm⁻^1^ range, associated with vibrations of its crystalline structure. The frequency band around 450 cm⁻^1^ is attributed to the internal deformation vibrations of the tetrahedral and octahedral units that constitute the four-element double ring (D^4^-R). The band with a width of approximately 650 cm⁻^1^ corresponds to the symmetric stretching vibrations of the Si–O and Al-O bonds, commonly referred to as T-bonds. At approximately 1000 cm⁻^1^, an intense band is observed, corresponding to the asymmetric stretching vibrations of the T(Si,Al)-O bonds, commonly observed in well-defined zeolitic structures. The band at ~ 1650 cm⁻^1^ is associated with the angular deformation of water molecules, indicating the presence of hydroxyl groups in the lattice. The broad band around 3500 cm⁻^1^ is characteristic of water molecules adsorbed within the cavities and porous channels of zeolite A, called zeolitic water. The water is weakly bound and can be removed by heat treatment without compromising the crystalline structure.

The geopolymer spectrum exhibits the most intense band in the 950–1100 cm⁻^1^ range, corresponding to the asymmetric stretching of T-O bonds (T = Si or Al), consistent with the main spectral signature of geopolymers. This band indicates the formation of a three-dimensional network of [SiO₄]₄⁻ and [AlO₄]₅⁻ tetrahedra linked by oxygen atoms, typical of the geopolymer matrix (Dembovska et al. 2018). In the 450–550 cm⁻^1^ region, a band corresponding to the bending vibrations of T–O–T bonds is observed, indicating the structural connectivity between the tetrahedral units. The bands in the 700–800 cm⁻^1^ range, although less intense, correspond to the symmetric stretching of Si–O–Si and Al–O–Si bonds. Additionally, the band at approximately 1650 cm⁻^1^ is associated with the angular deformation of physically adsorbed water (H–O–H) in the pores of the material. Finally, it was possible to attribute a broad band between 3200 and 3500 cm⁻^1^ to the stretching of O–H bonds. This indicates the presence of hydroxyl groups and adsorbed water, commonly found in fresh or partially cured geopolymer materials.

The spectrum of the compound under analysis shows similarities to the spectrum of zeolite A and the geopolymer. In the 400–700 cm⁻^1^ region, the overlapping signals between the composite and the zeolite indicate that the characteristic bands of the zeolite predominate, partially masking those of the geopolymer. This suggests that the crystalline structure of the zeolite was preserved during the synthesis process of the compound. In the 800–1700 cm⁻^1^ range, the signals of the compound show congruence with those of the geopolymer spectrum, corresponding to the T-O stretching modes of the aluminosilicate network and the carbonation of the material, as evidenced by a band between 1400 and 1500 cm⁻^1^ attributed to the formation of carbonate as a consequence of exposure to atmospheric carbon dioxide. Additionally, a slight narrowing of the broad band was observed around 3500 cm⁻^1^, which may indicate a reduced amount of free water in the final structure of the composite.

#### ***pH zero-point of charge (pH***_***pzc***_***)***

**Fig. 7:**
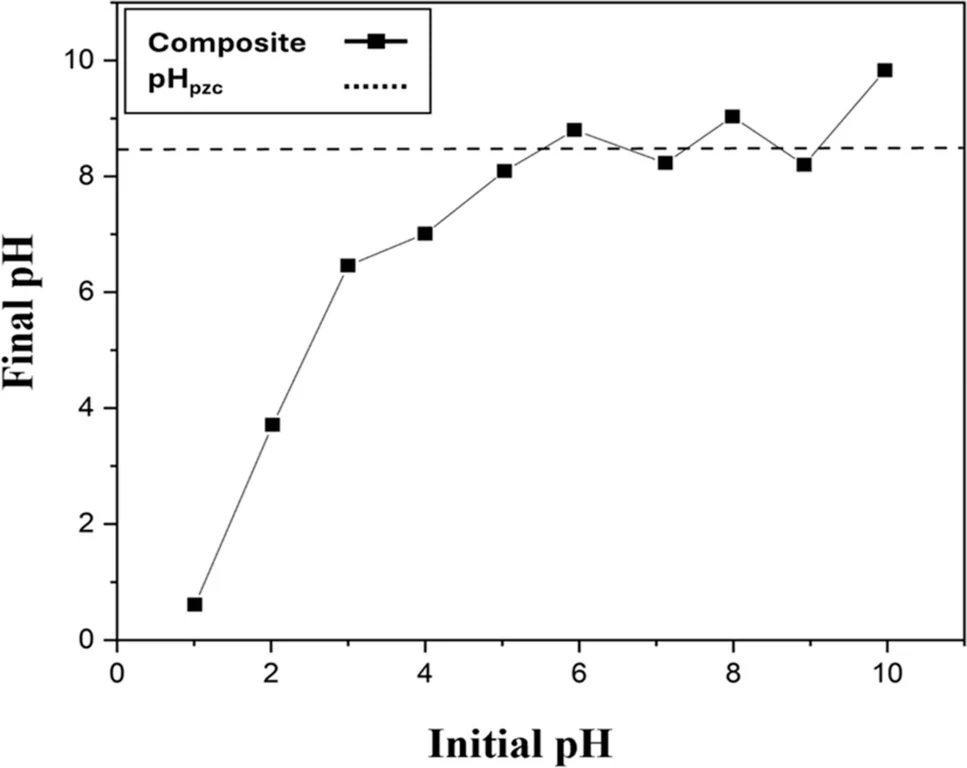
Graph for determining the pHpzc of the studied composite

The determination of the composite pHpzc was carried out through graphical analysis of the initial pH versus the final pH, as shown in Fig. 7. The pHpzc is identified on the plateau of this graph, calculated as the average of the final pH values that show minimal variation with increasing initial pH. The value obtained for the compound was 8.47. Similar materials obtained comparable results, presenting pHpzc values of 8.0 for natural zeolite (Ngeno et al. 2019), 7.7 for modified zeolite A (Derbe et al. 2025), and 7.5 for the geopolymer (Hermann et al. 2022).

#### Chemical composition characterization

The chemical composition of the Raw CFA and its non-magnetic fraction, the primary precursor utilized for the geopolymer-zeolite synthesis, was determined by X-ray fluorescence (XRF) and is presented in Table S1 (Supplementary Material). The analysis reveals that the non-magnetic fraction is predominantly composed of Si (48.41%) and Al (19.79%), confirming its suitability as a high-quality aluminosilicate source. Notably, the magnetic separation process resulted in a significant reduction in Fe content, from 29.60% in the raw ash to 8.32% in the precursor fraction. Regarding environmental safety, the XRF data demonstrate a negligible concentration of hazardous heavy metals in the synthesis fraction. Toxic elements of major regulatory concern, such as Lead (Pb), Cadmium (Cd), Selenium (Se), and Molybdenum (Mo), were not detected, while others like Arsenic (As) and Zinc (Zn) are present only in trace amounts (< 0.14%). In addition to the low initial concentration of these elements, the geopolymerization process itself is widely recognized for its ability to immobilize and stabilize trace contaminants within the aluminosilicate matrix (Ji & Pei 2020; Zhang & Liu 2013). This stabilization occurs through a combination of physical encapsulation and chemical binding within the three-dimensional network formed during synthesis. The resulting geopolymer structure acts as a barrier, significantly reducing the leaching potential of any trace metals present in the fly ash. These results indicate that the CFA precursor does not introduce significant levels of hazardous metals into the final composite, thereby minimizing concerns regarding secondary contamination and ensuring the material’s safety and reliability for aqueous remediation applications.

### Adsorption study

#### Characterization of the electroplating effluent and preparation of a synthetic solution

In the context of characterizing the electroplating effluent and preparing a synthetic solution, the anionic composition of the effluent was determined using ion chromatography (IC). On the other hand, the quantification of cations was carried out by flame atomic absorption spectrometry (FAAS). The results obtained are presented in Table 1. In summary, the effluent presented an acidic nature (pH 2), a value substantially lower than the limit established by Resolution No. 430/2011 of the National Environment Council (CONAMA—Brazil), which allows the discharge of industrial effluents only in the pH range of 5.0 to 9.0 (CONAMA, 2012).

**Table 1:** Composition of the galvanic effluent and the simulated synthetic solution

Ions	Concentration (mg·L^−1^)	
	**Real effluent**	**Synthetic solution**
**Chloride (Cl**^**−**^**)**	267.40 ± 7.05	270 ± 6.30
**Nitrate (NO**_**3**_^**−**^**)**	338.52 ± 8.21	340 ± 4.23
**Sulfate (SO**_**4**_^**2−**^**)**	898.14 ± 15.47	900 ± 11.34
**Copper (Cu**^**2+**^**)**	136.52 ± 2.76	52.5–315 ± 1.56
**Nickel (Ni**^**2+**^**)**	71.26 ± 1.84	49.5–297 ± 2.78
**Zinc (Zn**^**2+**^**)**	13.43 ± 0.32	62.6–335 ± 1.21

The solution for the synthetic effluent was prepared based on the data obtained in the characterization study of the real effluent, with the aim of simulating its matrix, especially in terms of ionic composition. The concentrations of metallic cations (Cu, Zn, and Ni) in the synthetic solution ranged from 50 to 300 mg·L⁻^1^. The pH of the synthetic solution was adjusted to approximately 5.0, a range in which the metallic species Cu^2+^, Ni^2+^, and Zn^2+^ are used to avoid interference from precipitation (da Silva et al. 2023; Santos et al. 2024).

#### Dosage test

**Fig. 8:**
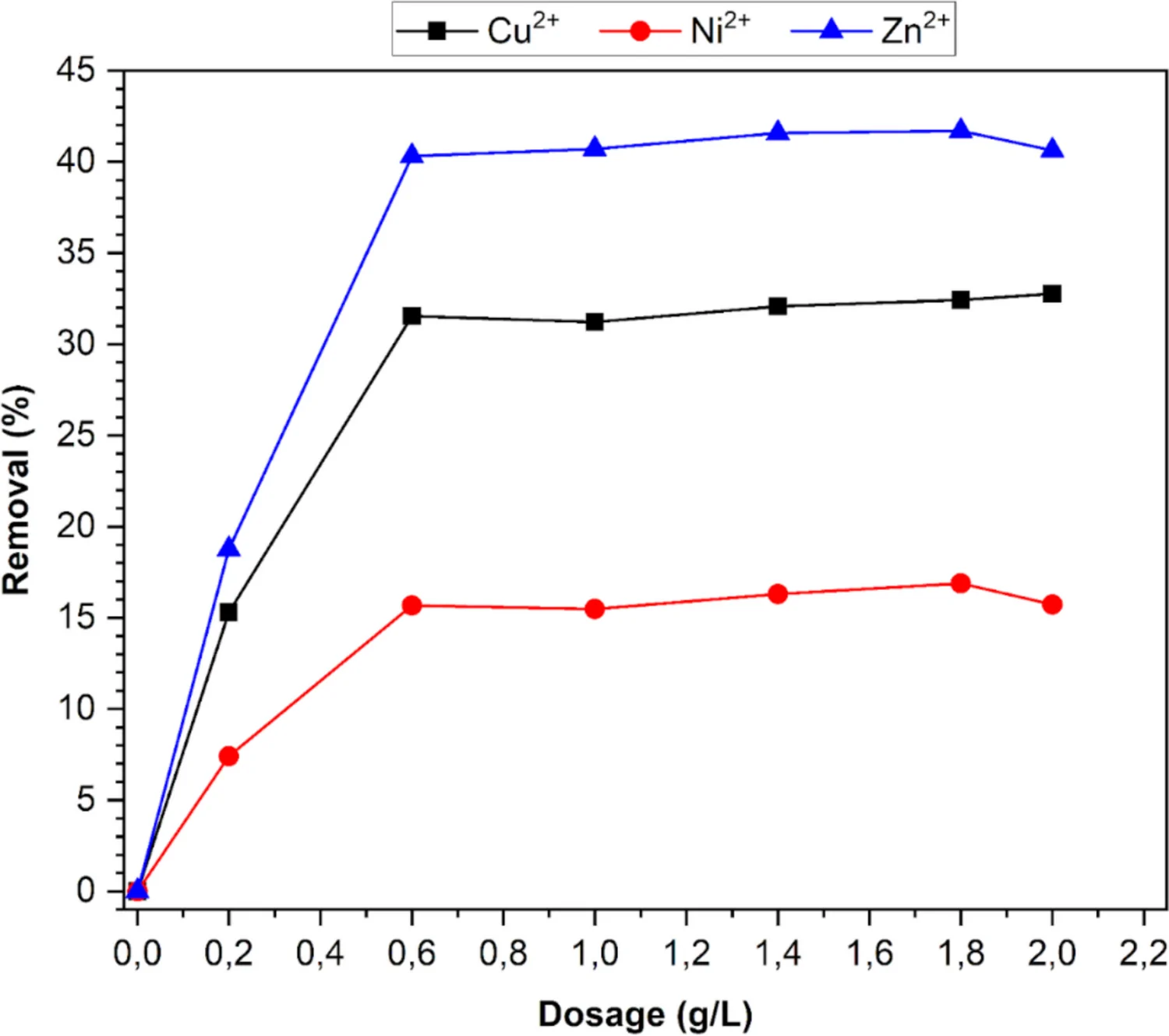
Effect of adsorbent dosage on the removal percentage of Cu^2^⁺, Ni^2^⁺, and Zn^2^⁺ (5 mmol, pH 5, 220 rpm, 25 °C, and 3 h)

The influence of adsorbent dosage on the removal efficiency of Cu^2+^, Ni^2+^, and Zn^2+^ is illustrated in Fig. 8. Increasing the dosage beyond 0.6 g L^−1^ did not yield a statistically significant improvement in cation uptake, suggesting that the available active sites reached a threshold of sufficiency relative to the initial adsorbate concentration (da Silva et al. 2023; Ngeno et al. 2019; Santos et al. 2024). At this stage, the adsorption process transitions from a site-limited regime to an adsorbate-limited regime, where the concentration gradient becomes the primary driving force (Santos et al. 2024; Yang et al. 2022). The plateau observed at higher dosages is attributed to the achievement of a solid–liquid equilibrium, where further increases in adsorbent mass do not enhance the removal percentage due to the near-exhaustion of available ions in the bulk solution. Consequently, a dosage of 0.6 g L^−1^ (30 mg of adsorbent per 50 mL) was identified as the optimal equilibrium point, balancing maximum removal efficiency with minimized material consumption. Therefore, this dosage was standardized for all subsequent kinetic and isothermal experiments.

#### Adsorption kinetics

**Fig. 9:**
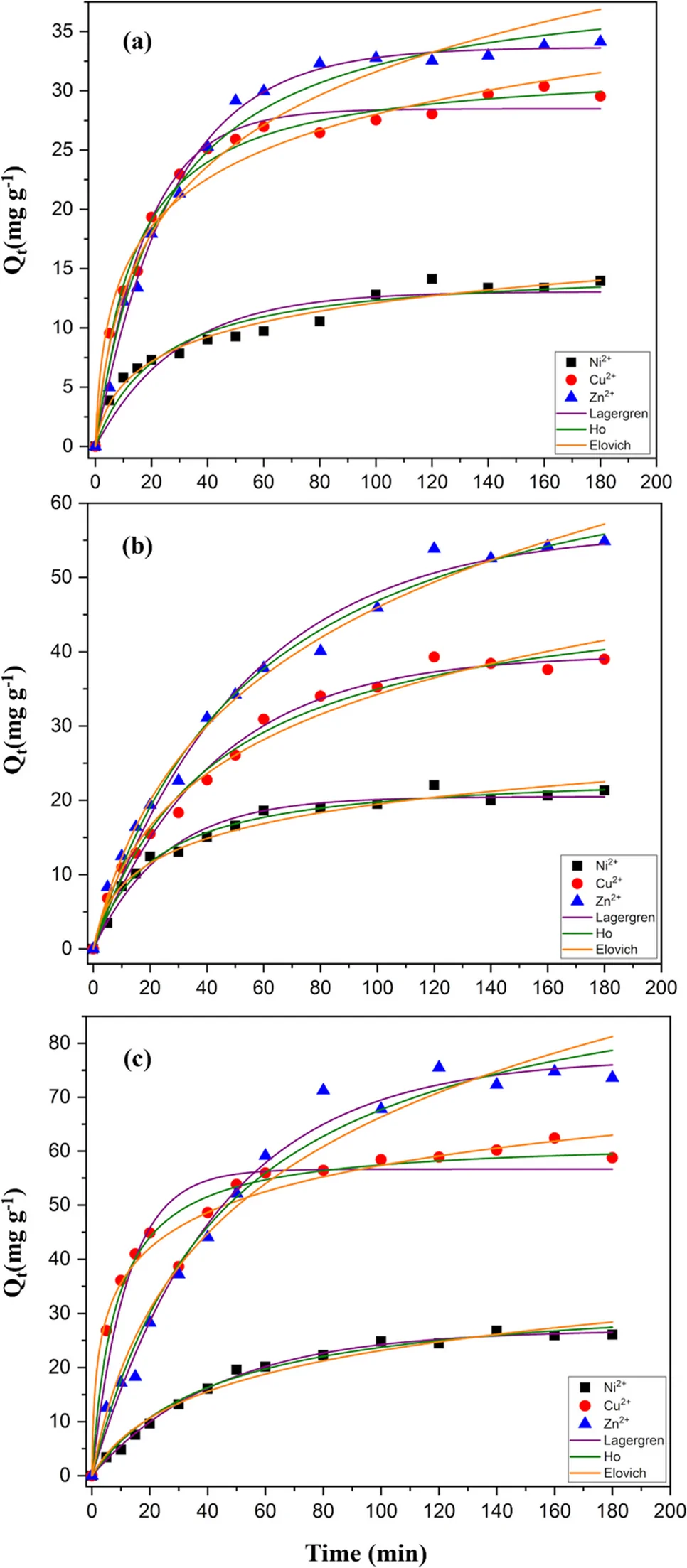
Kinetic adsorption profiles for Cu^2+^, Ni^2+^, and Zn^2+^ at initial concentrations of 0.8 (**a**) 2.5 (**b**) and 5.0 (**c**) mmol·L⁻^1^. pH = 5.0; 220 rpm; 29 °C

Kinetic evaluation is imperative in elucidating the adsorption mechanisms and ion behavior at the solid–liquid interface. In principle, the pseudo-first-order (PFO) equation is frequently the primary model employed to describe the adsorption rate in liquid systems (Al-Asadi et al. 2025; Y. Wang et al. 2024), exhibiting general applicability over the initial 20 to 30 min of interaction, where the constant *k*_*1*_ exhibits a tendency to decrease in conjunction with increasing initial concentration (Liu & Lian 2019). When the process follows a second-order mechanism, the Pseudo-Second Order (PSO) model is a more suitable approach. This is because *k*_*2*_ is influenced by operating conditions, such as pH and temperature, and serves as a metric for the time required to reach equilibrium (Ho & McKay 1999; Somsesta et al. 2020). Furthermore, the Elovich model is predicated on the assumption of energetically heterogeneous surfaces, rendering it optimal for the description of initial stages far from equilibrium, where the desorption rate is negligible. Based on these assumptions, the experimental kinetic profiles (Fig. 9) and adjusted parameters (Table 2) were analyzed for Cu^2+^, Zn^2+^ and Ni^2+^ ions. The statistical analysis of the data set revealed that the nonlinear fit exhibited superiority over the linear fit, as evidenced by the statistical criteria (*R*^2^, *χ*^2^, RMSE), which were found to be more effective in preserving the true distribution of errors and the accuracy of the physical parameters.

**Table 2:** Kinetic parameters obtained from the Lagergren (PFO), Ho (PSO), and Elovich Models for 0.8, 2.5, and 5.0 mmol·L⁻^1^, 220 rpm, pH = 5.0, 29 °C

		**Ni**^**2+**^ (mmol L^−1^)			**Cu**^**2+**^(mmol L^−1^)			**Zn**^**2+**^(mmol L^−1^)		
Models	**Parameters**	**0.85**	**2.5**	**5.0**	**0.85**	**2.5**	**5.0**	**0.85**	**2.5**	**5.0**
**Lagergren**	q_e_ (mg.g^−1^)	13.08	20.49	26.91	28.49	39.57	56.67	33.66	56.30	77.20
	k_1_ (min^−1^)	0.0337	0.0396	0.02305	0.0557	0.0237	0.08251	0.0365	0.0191	0.02304
	*R*^2^	0.9094	0.9777	0.9961	0.9812	0.9883	0.9096	0.9952	0.9867	0.9888
	*χ*^2^	1.629	1.080	0.361	1.588	2.154	27.01	0.663	4.771	8.334
**Ho**	q_e_ (mg.g^−1^)	15.18	23.92	34.05	32.18	49.78	62.19	39.92	73.47	98.79
	k_2_ (g·mg⁻^1^·min⁻^1^)	0.0028	0.002	0.0007	0.0023	0.0005	0.002	0.001	0.00024	0.00022
	*R*^2^	0.9525	0.9869	0.9946	0.9896	0.9886	0.9628	0.9876	0.9899	0.9806
	*χ*^2^	0.855	0.636	0.252	0.882	2.246	11.117	1.703	3.339	14.459
**Elovich**	α (mg·g⁻^1^·min⁻^1^)	1.29	2.101	0.974	6.24	1.65	38.51	2.78	1.67	2.846
	β (g·mg⁻^1^)	0.31	0.1908	0.1042	0.1659	0.0076	0.1046	0.1089	0.0048	0.0367
	*R*^2^	0.9759	0.9732	0.9784	0.9693	0.9822	0.9714	0.9636	0.9909	0.9681
	*χ*^2^	0.402	1.298	1.984	2.597	3.289	8.546	5.013	3.2640	23.798

Ni^2+^ demonstrated the slowest rate of kinetic behavior and the lowest adsorption capacity in comparison to the other metals under investigation. The kinetic behavior showed notable sensitivity to the initial concentration, with a transition between the three models: PFO at 0.8 mmol·L^−1^, PSO at 2.5 mmol·L^−1^, and Elovich at 5.0 mmol·L^−1^. This variation indicates that the nickel adsorption mechanism is complex and controlled by the progressive saturation of sites. The correlation between the linear and nonlinear models (Fig. S1) for the highest concentration (5.0 mmol·L^−^1) further supports the robustness of the parameters obtained, suggesting a predominance of external diffusion or physical adsorption processes in the initial stages.

The Cu^2+^ ion demonstrated rapid kinetics, particularly during the initial phases. At concentrations of 0.8 and 2.5 mmol·L^−1^, the data exhibited the greatest compatibility with the PSO model, thereby indicating that the adsorption rate is governed by chemisorption and contingent on the availability of active sites (Liu & Lian 2019). However, at the highest concentration (5.0 mmol·L^−1^), a change in behavior was observed. As shown in Fig. S2, the linear modelling approach indicated that the PSO model was the most suitable. However, the results of the nonlinear modelling indicated the Elovich model as the most representative, as evidenced by its high *R*^2^ and lower *χ*^2^ values. This finding is consistent with the observed physical behavior: copper exhibited an abrupt increase in adsorption capacity (high α in Table 2) followed by saturation, which is typical of heterogeneous surfaces with multilayer filling. In such cases, the Elovich model is superior in predicting kinetics far from equilibrium.

Zn^2+^ distinguished itself by exhibiting the highest equilibrium adsorption capacity values (*q*_*e*_) across all studied concentrations, surpassing both Cu^2^⁺ and Ni^2^⁺. Conversely, kinetic analysis revealed that Zn^2+^ presented the lowest rate constants (*k*_*2*_), indicating that, despite the material's high affinity and capacity for zinc, the saturation process is inherently slower. The PFO model provided the best fit for the experimental data at the extreme concentrations (0.8 and 5.0 mmol·L⁻^1^), in both non-linear and linear (at 5.0 mmol·L⁻^1^) fitting approaches (Fig. S3). This corroborates the applicability of the PFO model for describing initial adsorption stages or diffusion-controlled processes. Notably, the Elovich model was predominant exclusively at the intermediate concentration (2.5 mmol·L^−1^). These findings suggest that Zn^2+^ removal efficiency can be maximized under operationally optimized time conditions.

A comprehensive analysis reveals distinct competitive behavior among the ions. Notably, a capacity inversion phenomenon was observed between Cu^2+^ and Zn^2+^ at the highest concentration (5.0 mmol·L^−1^): while copper exhibits more intense adsorption during the initial 40 min, it is subsequently surpassed by zinc, which attains a superior equilibrium adsorption capacity (*q*_*e*_). Comparing these results with the literature reveals consistency in adsorption mechanisms, despite divergences in process kinetics. Kara et al. (2017), employing a metakaolin-based geopolymer, reported significantly shorter equilibrium times (40–50 min) compared to those observed in the present study (~ 100 min) (Kara et al. 2017). Nevertheless, their findings corroborate the affinity trend observed herein, reporting a good fit to the PSO model with *q*_*e*_ values of 65 mg·g^−1^ for Zn^2+^ and 31.7 mg·g⁻^1^ for Ni^2+^, thereby confirming the superior retention capacity for zinc relative to nickel. Similarly, Abdel Moamen et al. (2017), using a composite adsorbent, found that the experimental data were well described by both the PSO and intraparticle diffusion models. Their study also reported a behavioral inversion similar to that observed in this work, in which Zn^2^⁺ exhibited the highest initial rate, followed by Cu^2^⁺ and Ni^2^⁺ (Abdel Moamen et al. 2017). These comparisons underscore that although the geopolymer matrix in this study requires a longer contact time to reach equilibrium, it adheres to selectivity patterns (Zn > Cu > Ni) consistent with other aluminosilicate-based adsorbents and composites, where surface heterogeneity and initial concentration are determining factors in kinetic model selection. Furthermore, statistical analysis using one-way ANOVA (Table S2 – supplementary material) confirmed that the variations in adsorption capacity across different initial concentrations were statistically significant for all tested metals. The high *F*-values and low *p*-values (*p* < 0.05) indicate that the concentration is a critical factor affecting the removal process.

Overall, the kinetic modelling indicates a heterogeneous system in which the adsorption mechanism is not singular but rather depends on adsorbate concentration. The application of non-linear regression proved essential, preventing erroneous conclusions potentially arising from linearization (as exemplified by Cu^2^⁺ at 5.0 mmol·L⁻^1^), confirming that the adsorbent surface interacts in a complex and differentiated manner with each metal cation.

#### Adsorption isotherm

Isotherm analysis is critical for elucidating the equilibrium distribution of species and the nature of the adsorbent–adsorbate interaction (Al-Ghouti & Da’ana 2020; Ahmad et al. 2022). In this study, experimental data were fitted to the Langmuir (homogeneous monolayer), Freundlich (heterogeneous multilayer) and Sips (hybrid) models. Statistical evaluation (Table 3) demonstrated that the Sips non-linear regression provided the most robust fit for all cations (R^2^_adj_ > 0.96 and the lowest *χ*^2^ values). This confirms the energetic heterogeneity of the composite surface and its ability to overcome the limitations of two-parameter models at high concentrations.

**Table 3:** Parameters obtained from Langmuir, Freundlich, and Sips Model Fittings

Isotherm	Model Fittings	Parameters	Ni^2+^	Cu^2+^	Zn^2+^
**Langmuir**	**L**	**q**_**max**_	183.150	131.926	263.158
		**K**_**L**_	0.0022	0.0130	0.0043
		***R***^**2**^	0.9585	0.9758	0.9780
	**NL**	**q**_**max**_	97.820	119.525	236.185
		**K**_**L**_	0.00591	0.0174	0.0053
		***R***^**2**^	0.9581	0.9811	0.9672
		***χ***^**2**^	16.261	17.407	63.667
**Freundlich**	**L**	**K**_**F**_	1.382	10.955	3.276
		**1/n**	0.689	0.406	0.685
		***R***^**2**^	0.9015	0.8834	0.9486
	**NL**	**K**_**F**_	2.824	15.209	5.122
		**1/n**	0.551	0.341	0.597
		***R***^**2**^	0.9331	0.9435	0.9463
		***χ***^**2**^	25.977	47.343	94.797
**Sips**		**q**_**max**_	63.266	99.801	162.393
		**K**_**S**_	0.01198	0.0219	0.0104
		**1/n**	2.040	1.715	1.527
		**R**^**2**^_**adj**_	0.9759	0.9911	0.9686
		***χ***^**2**^	8.485	7.441	55.430

*L*, linear regression; *NL*, non-linear regression

**Fig. 10:**
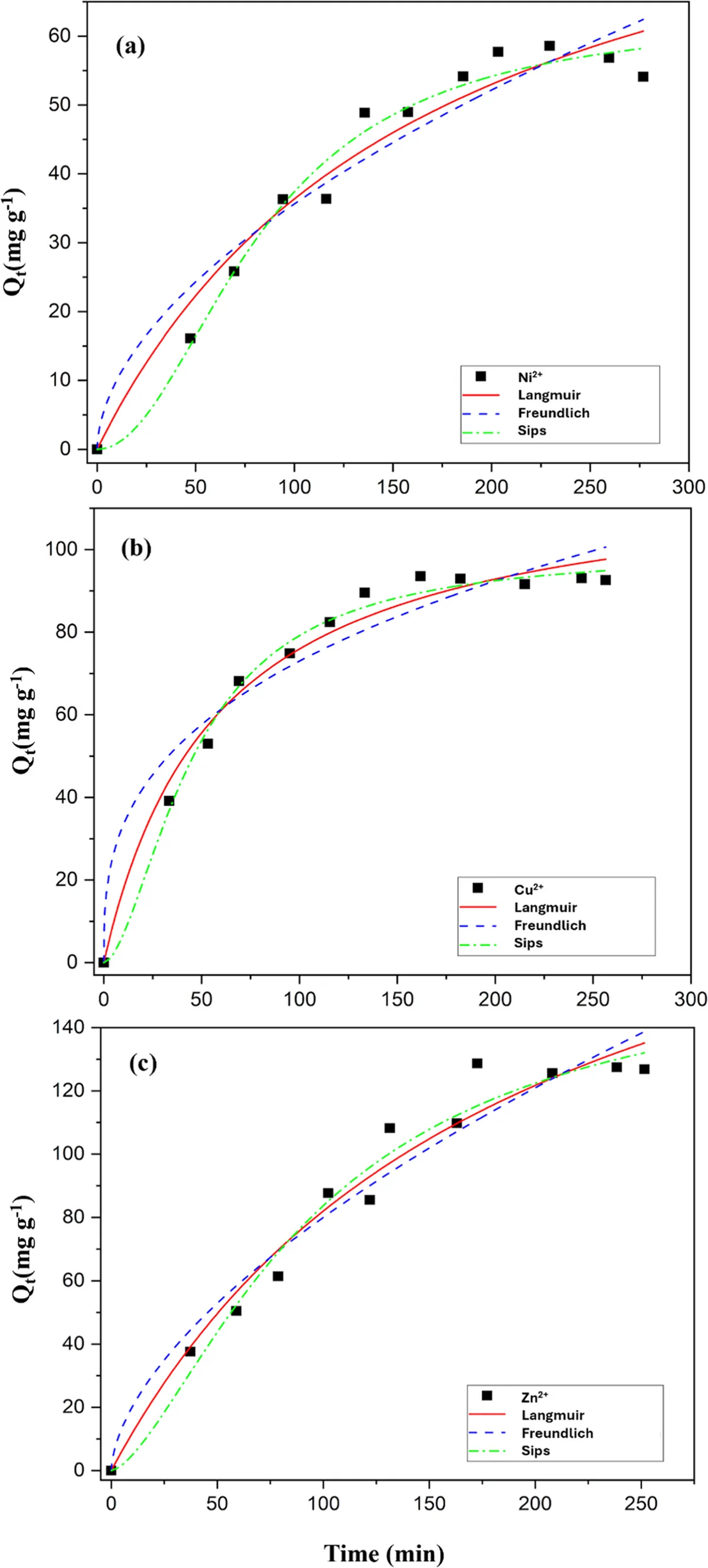
Adsorption Isotherm of Ni^2^⁺ (**a**), Cu^2^⁺ (**b**), and Zn^2^⁺ (**c**) Fitted to Langmuir, Freundlich, and Sips Models. pH = 5.0; 220 rpm; 29 °C

Ni^2+^ displayed the lowest adsorption capacity and a gradual growth curve. Mathematical analysis revealed a critical distortion inherent to linearization: the linear Langmuir model (Fig. S4) drastically overestimated *q*_*max*_ (183.1 mg·g^−1^) compared to the non-linear fit (97.8 mg·g^−1^) and the Sips model (63.3 mg·g^−1^), Fig. 10a. This underscores the inadequacy of linearization for this system, where the error distribution is non-normal. This behavior contrasts with Kara et al., who reported a higher affinity for Ni^2+^ in geopolymers (Kara et al. 2017); in the present study, the matrix showed selectivity in the order Zn > Cu > Ni for total capacity.

The isotherm profile for Cu^2+^ was characterized by a steep initial slope, indicating high affinity (elevated K_S_ and K_L_ values) and strong selectivity for the active sites. Saturation was reached at concentrations near 150 mg·L^−1^, Fig. 10b. In comparison, Abdel Moamen et al. (2017) reported saturation at higher concentrations (300 mg·L^−1^), suggesting that, while the current material may possess a lower density of available sites for copper, the interaction at existing sites is energetically favorable (Abdel Moamen et al. 2017). The discrepancy between linear and non-linear fittings was moderate; however, the non-linear approach effectively corrected for overestimations of affinity parameters.

Zn^2+^ exhibited the highest maximum adsorption capacity (*q*_*max*_) among the studied metals, achieving substantial values in the Sips model (162.4 mg·g^−1^) and non-linear Langmuir model (236.2 mg·g^−1^). Conversely, its affinity parameters (*K*) were lower than those of copper, implying that, while the material retains a larger quantity of zinc, the interaction force is less intense. Notably, Zn^2+^ showed the least distortion between linear and non-linear modelling (Figs. S6 and 10c), suggesting a more predictable adsorption behavior that is less susceptible to mathematical linearization errors.

In conclusion, the Sips model accurately established a maximum capacity order of Zn > Cu > Ni. This distinction is pivotal: zinc is removed in greater quantities at saturation, but copper is removed more efficiently at lower concentrations due to superior affinity. This selectivity profile contrasts with previous findings using hydrocarbons obtained by hydrothermal carbonization (Kara et al. 2017), where Ni^2^⁺ exhibited the highest adsorption capacity (180 mg·g^−1^). Furthermore, the saturation points for Cu^2+^ observed in this study (~ 150 mg·L^−1^) were lower than those reported in other studies (~ 300 mg·L^−1^) (Abdel Moamen et al. 2017), suggesting a distinct and potentially lower density of available active sites in the current composite matrix. Despite these quantitative differences, the application of non-linear regression proved indispensable for deriving robust physical parameters. It ensured that affinity and capacity values were determined without the distortions inherent to linearization, thereby confirming the adsorbent's predictable behavior and specific selectivity mechanisms.

Table 4 presents a comparison of the adsorption results from this study with data from the literature under different experimental conditions. Although several studies demonstrate the efficiency of adsorbents derived from industrial waste (Al-Harahsheh et al. 2015; Sarkar et al. 2017; Yang et al. 2022), most evaluate single-metal systems, without considering competition in multi-element matrices typical of real effluents. Furthermore, studies with geopolymer-zeolite composites applied to galvanic effluents containing multiple metals are still scarce (Luhar & Luhar 2022; Thakur et al. 2022). In this context, the proposed synthesis routes prove to be adaptable to alternative precursors, such as blast furnace slag and calcined clays (Provis 2014; Maged et al. 2023; Nodehi & Taghvaee 2022; Sarkar et al. 2017), maintaining the viability of ultra-low-cost adsorbents in a post-coal scenario.

**Table 4:** Comparison of adsorption results obtained in this study with literature data, considering different adsorbents and experimental conditions

Metal	Adsorbent	C_0_ (mg.L^−1^)	q_max_ (mg.g^−1^)	Safety/ Stability	Long-term Availability	Estimated cost	Reference
Cu	Chitosan/Histidine	20	2.36	Medium (Biodegradable)	Medium (High processing)	High	(Maia et al. 2020)
Zn	Chitosan/Histidine	20	2.35	Medium (Biodegradable)	Medium (High processing)	High	(Maia et al. 2020)
Cd	Biochar (Ginkgo leaves)	180	120.4	Medium (Leaching of organic matter)	High (Biomass sources)	Low—Medium	(Mei et al. 2025)
Cu	AGZA (Zeolite A)	6.4	1.84	High	Medium (Synthetic cost)	Medium	(França et al. 2021)
Ni	Polyethylene	5.86	0.059	Low (Microplastic risk)	High	Low	(de Vasconcelos Neto et al. 2024)
Cu	Polyethylene	4.4	0.191	Low (Microplastic risk)	High	Low	(de Vasconcelos Neto et al. 2024)
Cu	Fly Ash Geopolymer	100	152	Medium (Leaching risk)	Decreasing (Coal phase-out)	Ultra-low	(Al-Harahsheh et al. 2015)
Ni	LD slag geopolymer	150	85.29	Medium (Leaching risk)	High (Steel industry waste)	Low	(Sarkar et al. 2017)
Pb	Zeolite-geopolymer composite	200	446.73	High	Decreasing (Coal phase-out)	Ultra-low (Versatile precursors)	(Yang et al. 2022)
Ni/Cu/Zn	Zeolite-geopolymer composite	297/315/335	63.266/99.8/162.39	High	Decreasing (Coal phase-out)	Ultra-low (Versatile precursors)	This work

#### Adsorption mechanism

**Fig. 11:**
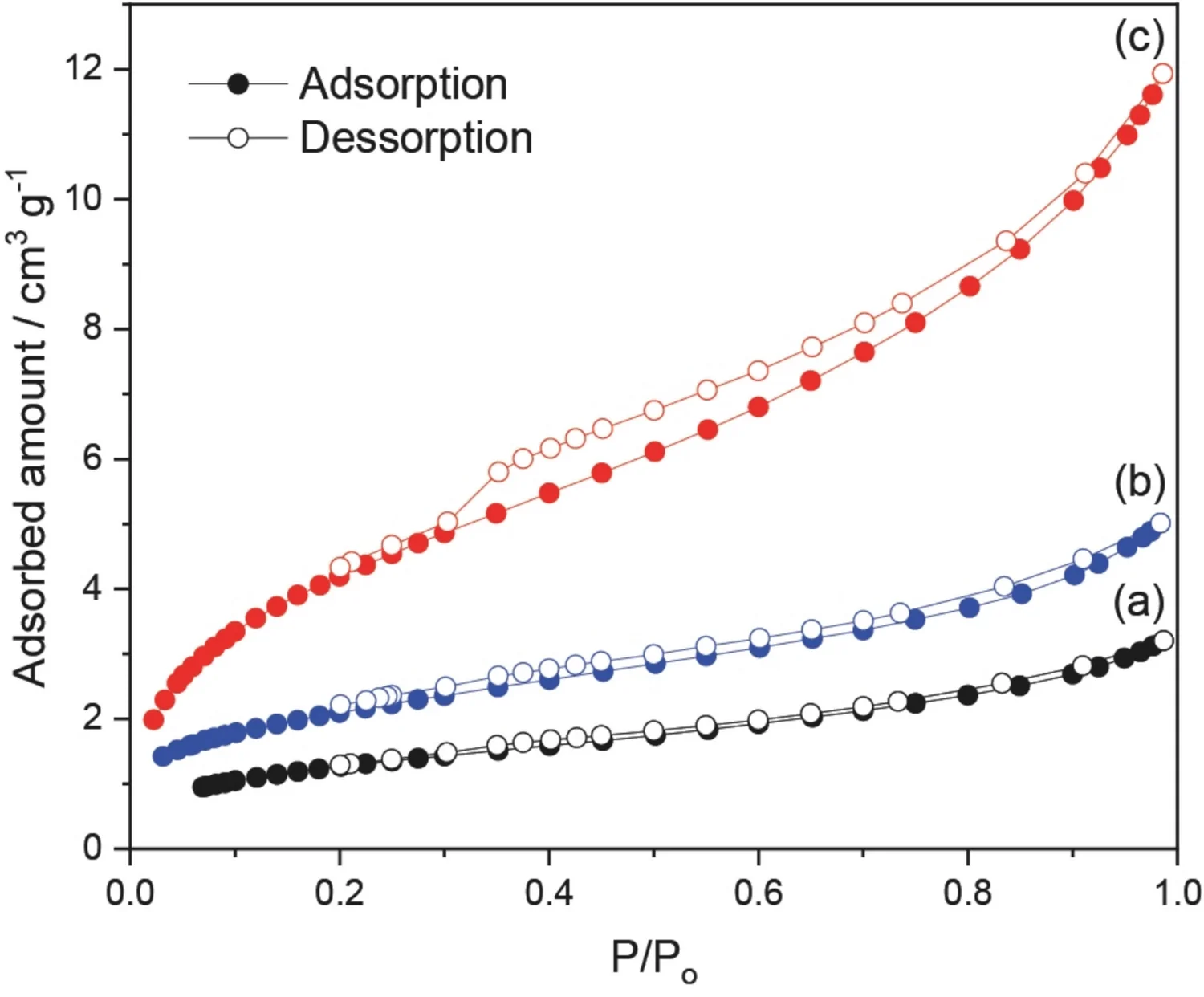
Ar adsorption/desorption isotherms of samples (**a**) Geopolymer, (**b**) Composite, and (**c**) Zeolite A

The textural properties obtained via argon adsorption/desorption isotherms (Fig. 11 and Table 5) are useful to understand the porous features of the samples and relate them to the capacity of removing metal ion from solution. The results demonstrate that the composite exhibits an intermediate porosity between the pure geopolymer and zeolite A. While the geopolymer matrix presented a low specific surface area (S_BET_ = 4 m^2^·g^−1^), the composite showed an increase to 7 m^2^·g^−1^, reflecting the successful formation of zeolite A crystalline phases (S_BET_ = 14 m^2^·g^−1^) within the amorphous framework. Despite the zeolite content in the composite being relatively low (ca. 3%), it contributes to a significant increase in pore volume (from 0.005 to 0.008 cm^3^·g^−1^), which is essential for enhancing the accessibility of hydrated metal cations to the internal active sites.

**Table 5:** Textural properties of the materials

Sample	S_BET_/m^2^g⁻^1^	Pore Volume/cm ^3^
Geopolymer	4	0.005
Composite	7	0.008
Zeolite A	14	0.018

This structural evolution directly supports the investigation of the ion exchange mechanism. To quantify this contribution, the release of sodium ions (Na^+^) was measured under specific test conditions (initial metal concentration of 5 mmol·L^−1^, pH 5.0, 220 rpm, 25 °C, for 3 h). The Na^+^ concentration increased significantly from 800 to 1112 mg·L^−1^ after contact (Fig. S7), confirming that sodium, a charge-balancing cation within the zeolitic cavities and geopolymeric channels, is displaced by Cu^2+^, Ni^2+^ and Zn^2+^ due to higher electrochemical affinity.

**Fig. 12:**
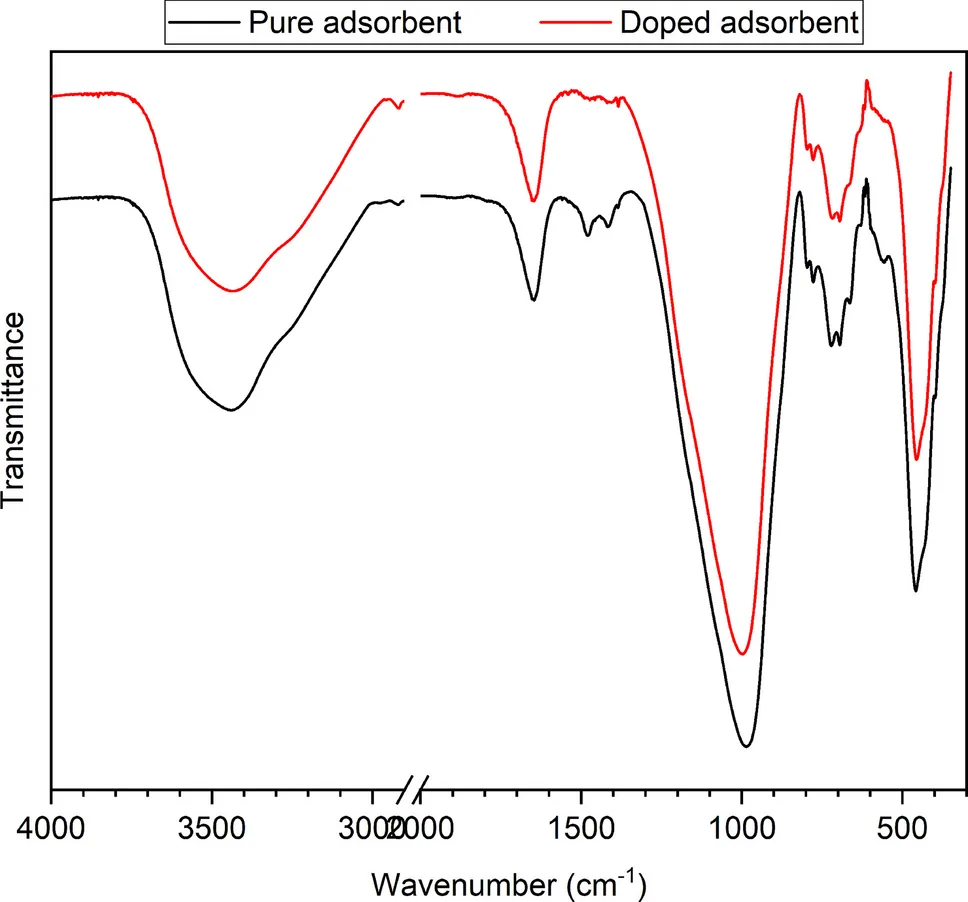
Comparative FTIR spectrum of the composite adsorbent before (black) and after (red) the adsorption process

The FTIR spectra, before and after adsorption (Fig. 12), further elucidate this process. The main characteristic bands of the aluminosilicate structure, particularly the Si–O–Al asymmetric stretching around 1000 cm^−1^, remain essentially unchanged, indicating that the geopolymer–zeolite framework was preserved during interaction with the metals.

The absence of new bands or significant shifts related to metal–oxygen bonding suggests that strong covalent complexation is not the primary driver. A minor spectral feature around 1500 cm^−1^, attributed to superficial carbonate species, disappeared after contact, likely due to dissolution in the aqueous medium. The minimal spectral changes, combined with the substantial Na^+^ release and improved textural accessibility, confirm that the adsorption process is governed predominantly by ion exchange. The porous geopolymeric matrix acts as a scaffold, facilitating ion transport, while the dispersed zeolite A phases provide the high-affinity exchange sites. This synergistic mechanism allows the composite to outperform the base geopolymer, aligning with findings that highlight ion exchange as a robust pathway for heavy metal remediation in aluminosilicate systems (Santos et al. 2024).

## Conclusion

This study successfully demonstrated the synthesis of a geopolymer–zeolite A composite using coal fly ash as a precursor, resulting in a multifunctional material that preserves the essential structural features of both phases. The coexistence of an amorphous geopolymer matrix and crystalline zeolite A structures provides a heterogeneous surface with adsorption sites of varying energies, which is fundamental for treating complex, multi-elemental matrices. In batch adsorption tests using simulated electroplating effluents, the composite exhibited distinct selectivity for Cu^2^⁺, Ni^2^⁺, and Zn^2^⁺. Copper exhibited the fastest initial adsorption kinetics, consistent with its smaller solvation radius and high affinity for the material's functional groups. Conversely, zinc reached a remarkable maximum adsorption capacity (q_max_) 162.39 mg·g^−1^ according to the Sips model, confirming the material's high performance even under elevated metal loadings. The identified ion-exchange mechanism, evidenced by the release of sodium ions, underscores the specific contribution of the zeolite A phase to the overall remediation process. Beyond its adsorptive performance, this study highlights the zeolite-geopolymer composite as a promising candidate for the valorization of legacy coal fly ash, aligning with circular-economy principles by transforming an industrial liability into a high-value environmental solution. While the results under controlled laboratory conditions are highly encouraging, it is acknowledged that the transition to industrial-scale application requires further investigation. Future studies should focus on the material’s long-term leaching stability, performance in real-world industrial wastewater, and the feasibility of regeneration cycles to fully establish its technical and regulatory viability.

## Supplementary information

Below is the link to the electronic supplementary material.

Supplementary file1 (DOCX 943 kb)

## Data Availability

The datasets generated and/or analyzed during the current study are available from the corresponding author on reasonable request.
