# Supplementary material for: Study of the potential of composite material based on zeolite and geopolymer as an adsorbent of toxic metals
Source: Environ Sci Pollut Res Int. 2026 Apr 29;33(33):16545–63. doi: 10.1007/s11356-026-37751-7 (PMC13649859; doi:10.1007/s11356-026-37751-7)
Supplement: Supplementary file 1 [file 11356_2026_37751_MOESM1_ESM.docx]

**Supplementary material**

**Study of the potential of composite material based on zeolite and geopolymer as an adsorbent of toxic metals**

Ricardo Jadson da Silva Nascimento ^1^, Inácio Cruz de Loiola^1^, João Vitor Torres Sousa^1^, Vicente de Oliveira Sousa Neto ^2^, Luiz Thiago Vasconcelos da Silva ^1^, *Ronaldo Ferreira do Nascimento ^1^

^1^Laboratory of Trace Analysis-LAT/Department of Analytical and Physical Chemistry, Federal University of Ceara, Fortaleza, Brazil

^2^Laboratory of Study and Research in Pollutants Removal by Adsorption-LERPAD/Department of Chemistry, State University of Ceara, Quixadá, Brazil

*Corresponding author (ronaldo@ufc.br)

**Figure S1** – Linear Kinetic Models for Ni²⁺ at 5 mmol·L⁻¹: (a) Lagergren (Pseudo-First Order - PFO); (b) Ho (Pseudo-Second Order - PSO); (c) Elovich. pH = 5,0; 220 rpm; 29 ºC.

**Figure S2** – Linear Kinetic Models for Cu²⁺ at 5 mmol·L⁻¹: (a) Lagergren (Pseudo-First Order - PFO); (b) Ho (Pseudo-Second Order - PSO); (c) Elovich. pH = 5,0; 220 rpm; 29 ºC.

**Figure S3** – Linear Kinetic Models for Zn²⁺ at 5 mmol·L⁻¹: (a) Lagergren (Pseudo-First Order - PFO); (b) Ho (Pseudo-Second Order - PSO); (c) Elovich. pH = 5,0; 220 rpm; 29 ºC.

**Figure S4 –** Linearized Adsorption Isotherm Models for Ni²⁺: (a) Langmuir and (b) Freundlich. pH = 5.0; 220 rpm; 29 ºC.

**Figure S5 –** Linearized Adsorption Isotherm Models for Cu²⁺: (a) Langmuir and (b) Freundlich. pH = 5.0; 220 rpm; 29 ºC.

**Figure S6 –** Linearized Adsorption Isotherm Models for Zn²⁺: (a) Langmuir and (b) Freundlich. pH = 5.0; 220 rpm; 29 ºC.

**Figure S7** – Ion Exchange effect.

**Table S1 –** Elemental analysis (wt.%) of the starting coal fly ash (CFA) and the purified, non-magnetic fraction used for geopolymer–zeolite synthesis, obtained via X-ray fluorescence (XRF).

| **Element** | **Fly ash (%)** | **Non-magnetic fraction (%)** |
| --- | --- | --- |
| Al | 13.200 | 19.796 |
| Si | 38.935 | 48.416 |
| Fe | 29.601 | 8.322 |
| K | 5.217 | 5.296 |
| Na | - | 1.811 |
| Mg | - | 1.217 |
| Ca | 8.033 | 8.974 |
| S | 0.822 | 0.321 |
| Ti | 2.635 | 3.103 |
| Rb | 0.087 | - |
| Mn | 0.171 | 0.262 |
| Sr | 0.814 | 1.018 |
| Ni | 0.141 | - |
| As | 0.113 | 0.136 |
| Zn | 0.158 | 0.135 |
| Pb | - | - |
| Mo | - | - |
| Br | - | - |
| Se | - | - |
| P | - | 0.285 |
| Co | - | 0.109 |
| Cd | - | - |
| Cl | 0.069 | - |
| **Total** | 99.99 | 99.30 |

**Table S2 –** One-way ANOVA for Cu²⁺, Ni²⁺ and Zn²⁺ adsorption capacity (qₑ).

| **One-way ANOVA for Cu²⁺ adsorption capacity (qₑ)** | | | | | | |
| --- | --- | --- | --- | --- | --- | --- |
| **Source of variation** | **SS** | | **df** | **MS** | **F-value** | **p-value** |
| **Between concentrations** | 1300.35 | | 2 | 650.17 | 93.86 | 2.97 × 10⁻⁵ |
| **Within groups (error)** | 41.56 | | 6 | 6.93 |  |  |
| **Total** | 1341.91 | | 8 |  |  |  |
| **One-way ANOVA for Ni²⁺ adsorption capacity (qₑ)** | | | | | | |
| **Source of variation** | **SS** | **df** | | **MS** | **F-value** | **p-value** |
| **Between concentrations** | 307.05 | 2 | | 153.53 | 36.89 | 4.25 × 10⁻⁴ |
| **Within groups (error)** | 24.97 | 6 | | 4.16 |  |  |
| **Total** | 332.02 | 8 | |  |  |  |
| **One-way ANOVA for Zn²⁺ adsorption capacity (qₑ)** | | | | | | |
| **Source of variation** | **SS** | | **df** | **MS** | **F-value** | **p-value** |
| **Between concentrations** | 2311.60 | | 2 | 1155.80 | 186.03 | 4.00 × 10⁻⁶ |
| **Within groups (error)** | 37.28 | | 6 | 6.21 |  |  |
| **Total** | 2348.88 | | 8 |  |  |  |
